# Mapping HLA-A2, -A3 and -B7 supertype-restricted T-cell epitopes in the *ebolavirus* proteome

**DOI:** 10.1186/s12864-017-4328-8

**Published:** 2018-01-19

**Authors:** Wan Ching Lim, Asif M. Khan

**Affiliations:** 1 Centre for Bioinformatics, Perdana University School of Data Sciences, 43400 Serdang, Selangor Malaysia; 2 Department of Pharmacology and Molecular Sciences, The Johns Hopkins University School of Medicine, 725 North Wolfe Street, Baltimore, MD 21205 USA

**Keywords:** *Ebolavirus*, T-cell epitope, HLA supertype, Antigenic diversity

## Abstract

**Background:**

*Ebolavirus* (EBOV) is responsible for one of the most fatal diseases encountered by mankind. Cellular T-cell responses have been implicated to be important in providing protection against the virus. Antigenic variation can result in viral escape from immune recognition. Mapping targets of immune responses among the sequence of viral proteins is, thus, an important first step towards understanding the immune responses to viral variants and can aid in the identification of vaccine targets. Herein, we performed a large-scale, proteome-wide mapping and diversity analyses of putative HLA supertype-restricted T-cell epitopes of *Zaire ebolavirus* (ZEBOV), the most pathogenic species among the EBOV family.

**Methods:**

All publicly available ZEBOV sequences (14,098) for each of the nine viral proteins were retrieved, removed of irrelevant and duplicate sequences, and aligned. The overall proteome diversity of the non-redundant sequences was studied by use of Shannon’s entropy. The sequences were predicted, by use of the NetCTLpan server, for HLA-A2, -A3, and -B7 supertype-restricted epitopes, which are relevant to African and other ethnicities and provide for large (~86%) population coverage. The predicted epitopes were mapped to the alignment of each protein for analyses of antigenic sequence diversity and relevance to structure and function. The putative epitopes were validated by comparison with experimentally confirmed epitopes.

**Results & discussion:**

ZEBOV proteome was generally conserved, with an average entropy of 0.16. The 185 HLA supertype-restricted T-cell epitopes predicted (82 (A2), 37 (A3) and 66 (B7)) mapped to 125 alignment positions and covered ~24% of the proteome length. Many of the epitopes showed a propensity to co-localize at select positions of the alignment. Thirty (30) of the mapped positions were completely conserved and may be attractive for vaccine design. The remaining (95) positions had one or more epitopes, with or without non-epitope variants. A significant number (24) of the putative epitopes matched reported experimentally validated HLA ligands/T-cell epitopes of A2, A3 and/or B7 supertype representative allele restrictions. The epitopes generally corresponded to functional motifs/domains and there was no correlation to localization on the protein 3D structure. These data and the epitope map provide important insights into the interaction between EBOV and the host immune system.

**Electronic supplementary material:**

The online version of this article (10.1186/s12864-017-4328-8) contains supplementary material, which is available to authorized users.

## Background

Ebola virus disease (EVD) or Ebola haemorrhaging fever, although not as commonly spread as influenza, is more often deadly once contracted, and thus, making it one of the most fatal diseases encountered by mankind [1]. The recent 2014 Ebola epidemic was the largest in recorded history. According to the United States Centers for Disease Control and Prevention, there were a total of 11,965 confirmed Ebola cases, including 6446 fatalities as of July 8, 2015 [2]. Although the epidemic primarily affected West Africa, it has the potential to spread to other parts and outside of Africa. The causative agent of the disease is the *Ebolavirus* (EBOV), a member of the *Filoviridae* family. Ever since the EBOV was first discovered in 1976 [1], EVD has claimed the lives of many, especially during the several outbreaks throughout the years. Among the five species of the EBOV, *Zaire ebolavirus* (ZEBOV) is observed to account for the highest mortality rate among EVD patients [3, 4]. Although vaccine studies had over the years achieved milestones in identifying trial candidates at various phases [5], an effective vaccine against EBOV for human usage is currently not publicly available.

The adaptive immune response (both humoral and cellular) plays a key role in protection against viral pathogens [6]. The cellular T-cell responses, involving both the CD4^+^ and CD8^+^ T cells, have been implicated to be important in providing protection against the EBOV [7]. Notably, the EBOV proteins GP and NP have been known to stimulate T-cell responses. Immunization with adenoviral vectors (AdV) encoding the GP and NP stimulated the induction of effective protection in nonhuman primates [8]. T-cell epitopes, immunogenic peptides presented by the human leukocyte antigen (HLA) molecules as targets of cellular immune responses, are critical elements for protection against pathogens. CD4^+^ T-cell epitope presentation is necessary for humoral antibody production by B cells during the breach of the immune system. Sequence changes in the epitopes (antigenic variation), even of a single amino acid, can result in viral escape from immune recognition [9–11]. Therefore, mapping targets of immune responses among the sequence of viral proteins is an important first step towards understanding the immune responses to viral variants and can aid in the identification of vaccine targets.

A challenge in identifying T-cell epitopes is the high polymorphism of HLA, recognised as the most polymorphic human loci [12]. As of January 2016, there were more than 14,000 HLA alleles reported for the human population [13]. Notably, it was discovered that certain HLA molecules share similar peptide binding specificity and can be classified into groups termed as HLA supertypes [14]. T-cell epitopes promiscuous to multiple alleles of a supertype are the best targets to map and study because they are applicable to a large proportion of the human population [15] by providing an extensive coverage across different ethnicities [14]. In fact, a cocktail vaccine with peptides relevant to just three of the HLA supertypes (A2, A3 and B7) can cover ~86% of the human population [14]. Although many experimentally confirmed HLA ligands of ZEBOV have been reported, only a limited number of human T-cell epitopes are known [16]. Bioinformatics tools for predictions of HLA-binding peptides have been proven to minimize the cost and time for experimental T-cell epitope mapping [17]. These tools utilize a plethora of advanced algorithms for the prediction of HLA binding peptides [17–20], and allow the prediction for a wide range of HLA alleles. Prediction in the context of HLA supertypes is offered by a number of the tools, such as Hotspot Hunter [21], MAPPP [22], MULTIPRED2 [23], PEPVAC [24], and NetMHC [25], among others. Earlier studies had elucidated putative T-cell epitopes of individual EBOV proteins [26–30] by use of bioinformatics tools, such as SYFPEITHI [26, 29], BIMAS [28, 29], IEDB [26, 29], NetMHCcons [30], NetChop [30], NetCTL [27], NetCTLpan [29], NetMHCpan [26] and NetMHCIIpan [30]. However, the identification and analyses of T-cell epitopes from the complete ZEBOV proteome, especially in the context of HLA-supertype restriction and application of prediction on multiple aligned sequences has thus far remained limited; existing studies [26, 27, 31–33] either do not cover all the proteins or all available sequences of the proteins, with limited or no antigenic diversity analysis across the sequences.

Herein, we applied a computational approach to map and analyse putative HLA supertype-restricted T-cell epitopes of ZEBOV, the most pathogenic species among the EBOV family. The epitopes were predicted for all representative HLA alleles of supertypes A2, A3 and B7, which provide a high coverage of the human population and are applicable to the major ethnic groups including those from Africa (Caucasian, 83%; North American Blacks, 86.1%; Japanese, 87.5%; Chinese, 88.4%; Hispanic, 86.3%; African, 75.1%) [14, 34]. The diversity of the entire ZEBOV proteome, including the mapped epitopes (antigenic diversity) were determined and analysed. Additionally, the putative epitopes were compared with reported experimentally confirmed epitopes.

## Materials and methods

### Data collection

Ebola protein sequences (as of April 2016) were collected through the National Center for Biotechnology Information (NCBI) Taxonomy database [35] by use of the taxonomy identifier (ID) “186538” for *Zaire ebolavirus* species. This species has been observed to have the highest mortality among human population compared to the other species of the virus, namely *Sudan ebolavirus*, *Tai Forest ebolavirus* (originally *Côte d’Ivoire ebolavirus*), *Reston ebolavirus*, and *Bundibugyo ebolavirus* [36].

### Data processing: Cleaning and grouping

The ZEBOV genome consists of a single-stranded negative sense RNA, which is approximately 19 kb long, and encodes for nine proteins, namely glycoprotein (GP), non-structural soluble glycoprotein (sGP), second secreted glycoprotein (ssGP), nucleoprotein (NP), virion structural proteins (VP40, VP35, VP30, VP24), and RNA-dependent RNA polymerase (L). Reference sequences for each protein were obtained from the NCBI RefSeq Database [37] (NP: NP_066243.1; VP35: NP_066244.1; VP40: NP_066245.1; GP: NP_066246.1; sGP: NP_066247.1; ssGP: NP_066248.1; VP30: NP_066249.1; VP24: NP_066250.1; L: NP_066251.1). The protein sequences downloaded through the NCBI Taxonomy Database were used to build a local searchable BLAST [38] database. Each RefSeq reference protein sequence was used as a query for a BLAST search against the database to extract and group sequences of the protein. The BLAST results were manually inspected to remove irrelevant hits, which included non-EBOV fragments and non-ZEBOV protein sequences. Moreover, the inspection revealed that some sequences were polyprotein, which included sequences of the immediate neighbouring protein. For example, a number of the NP sequences contained fragments of VP35 and vice versa. Thus, VP35 fragments in NP sequences were removed and added to the collection of VP35 dataset, and vice versa. Full-length and partial duplicate sequences (100% identity) of each protein were removed from the dataset. The related GP protein sequences (GP, sGP and ssGP) were split into two parts with respect to the length: the first 295 amino acids were a common region, and thus, were referred to as the “Pre-295 All GP”; the remaining part differed in length between the three GP proteins due to different reading frame translation [39], and thus were accordingly referred to as “Post-295 GP”, “Post-295 sGP”, and “Post-295 ssGP” (was not studied as it consisted of only less than 5 amino acids). All the GP parts were collectively referred to as “All GP”. Protein sequences were aligned by use of ClustalOmega [40] (default settings) and manually inspected for misalignments.

### Diversity of ZEBOV proteome

The diversity of ZEBOV proteome was measured by use of Shannon’s entropy [41–43]. This was done for overlapping nonamers (1–9, 2–10, etc.) of the aligned sequences of each protein. Window size of nine was chosen for immunological applications [41, 42]: it is the typical length of HLA class I T-cell epitopes and the core length of the HLA class II epitopes. The entropy of a given nonamer position (*x*) in a sequence alignment is defined as:

$$ \mathrm{H}\left(\mathrm{x}\right)=-\sum \limits_{\mathrm{i}=1}^{\mathrm{n}\left(\mathrm{x}\right)}\mathrm{p}\left(\mathrm{i},\mathrm{x}\right){\log}_2\mathrm{p}\left(\mathrm{i},\mathrm{x}\right) $$

where *p(i, x)* represents the probability of a particular nonamer peptide *i* at position *x*. The entropy value increases as the total number of peptides, *n(x)*, present at the position *x* increases. The entropy value drops in cases where a certain peptide is dominant at the position *x*. Highly conserved regions yield low entropy values, approaching zero when there is a complete (100%) conservation. Only nonamer peptides of valid amino acids at position *x* were analyzed. Nonamers containing gaps were ignored in the calculation of the entropy. The entropy bias is inversely proportional to the sample size N [44], where as N increases to ∞, bias reduces, approaching zero. Given the finite number of sequences studied, a correction to the estimation of the entropy value at a nonamer position *x* was performed by randomly sampling N into smaller datasets of sequences. Entropy values were computed for all the datasets and then plotted against 1/N. Extrapolating the linear regression of the entropy values by using line of best fit to 1/N of zero will yield the estimated entropy at position *x* when N is large (approaching ∞).

### Identification of known EBOV HLA ligands or T-cell epitopes

Experimentally validated MHC ligands or T-cell epitopes for EBOV were obtained from the Immune Epitope Database and Analysis Resource (IEDB) (as of January 2017). All linear human T-cell epitopes relevant to EBOV from positive assays, such as T cell assays or HLA (human MHC) ligand assays, were downloaded, analysed, and compared with the predicted epitopes.

### Prediction of T-cell epitopes within the ZEBOV proteome

T-cell epitopes were predicted by use of a local copy of NetCTLpan 1.1 [45], with default parameters. The method integrates prediction of various stages of the antigen processing pathway: MHC class I peptide binding (by use of artificial neural networks; ANN), proteasomal C terminal cleavage (ANN) and TAP transport efficiency (weight matrix). The predictive performance of the NetCTLpan has been reported to outperform other state-of-the-art class I epitope prediction methods [46]. Prediction was done for peptide length of nine and for the representative alleles of the supertypes A2 (A*0201, A*0202, A*0203, A*0204, A*0205, A*0206, A*0207, A*0214, A*0217, A*6802, A*6901), A3 (A*0301, A*1101, A*3101, A*3301, A*3303, A*6601, A*6801, A*7401), and B7 (B*0702, B*0703, B*0705, B*1508, B*3501, B*3503, B*4201, B*5101, B*5102, B*5103, B*5301, B*5401, B*5501, B*5502, B*5601, B*6701, B*7801). Since a peptide may not be predicted as an epitope for every representative allele of a supertype, a 50% cut-off was set to determine a putative supertype-specific epitope (i.e. peptides predicted as epitopes for at least half of the representative alleles were selected). The A2, A3 and B7 supertypes were chosen because they are reported to provide a wide population coverage (86%) [14] and are relevant to African and other ethnicities. An epitope frequency table for each protein was created from the non-redundant list of predicted supertype-specific epitopes. These were then mapped to the respective protein alignments for an overview of the epitope distributions and identification of possible hotspots (a cluster of at least three or more overlapping epitopes) [47]. Putative epitopes that were interleaved by one or more gaps in the alignment or those that contained unknown amino acid “X” were excluded from any analysis.

### Antigenic diversity of putative supertype-specific T-cell epitopes

All sequences at each of the mapped, putative epitope alignment positions were extracted and analysed for antigenic diversity. The different epitope and non-epitope sequences at the position and their individual frequency were noted. The entropy of the position, individual frequency of the putative epitope sequences versus the non-epitope sequences and the amino acid substitutions between the putative epitope sequences at the same position were assessed.

### Functional and structural analyses of the predicted epitopes

The known and putative structural and functional properties of the predicted epitopes were searched in the literature and by use of Pfam [48], InterPro [49], and CDD databases [50]. Where possible, the sequences were mapped onto the three-dimensional (3-D) structures of available ZEBOV antigen in the PDB database [51] by use of Molsoft ICM Browser.

## Results

### ZEBOV protein sequence dataset

A total of 14,113 *Zaire ebolavirus* protein sequences were retrieved from the NCBI Taxonomy Database. This included 19 non-ZEBOV sequences, which were removed, and four polyprotein sequences (see Methods), which provided additional four sequences, and thus, resulting in a total of 14,098 relevant sequences. The removal of duplicate sequences reduced the number of sequences by ~91% to 1272: 208 (NP), 125 (VP35), 109 (VP40), 353 (GP, sGP, ssGP), 66 (VP30), 69 (VP24) and 342 (L) (Table 1). VP30 had the most redundant sequences (~96%), while L had the least (~80%).

**Table 1:** Number and distribution of redundant and non-redundant ZEBOV sequences

Protein	Redundant sequences	Non-redundant sequences	Sequence reduction (%)^a^
NP	1714	208	87.86
VP35	1753	125	92.86
VP40	1714	109	93.64
All GP	3827	353	90.78
VP30	1665	66	96.03
VP24	1715	69	95.97
L	1710	342	80.00
Total	14,098	1272	90.97

^a^Rounded to two decimal places

### Diversity of ZEBOV proteome

**Fig. 1:**
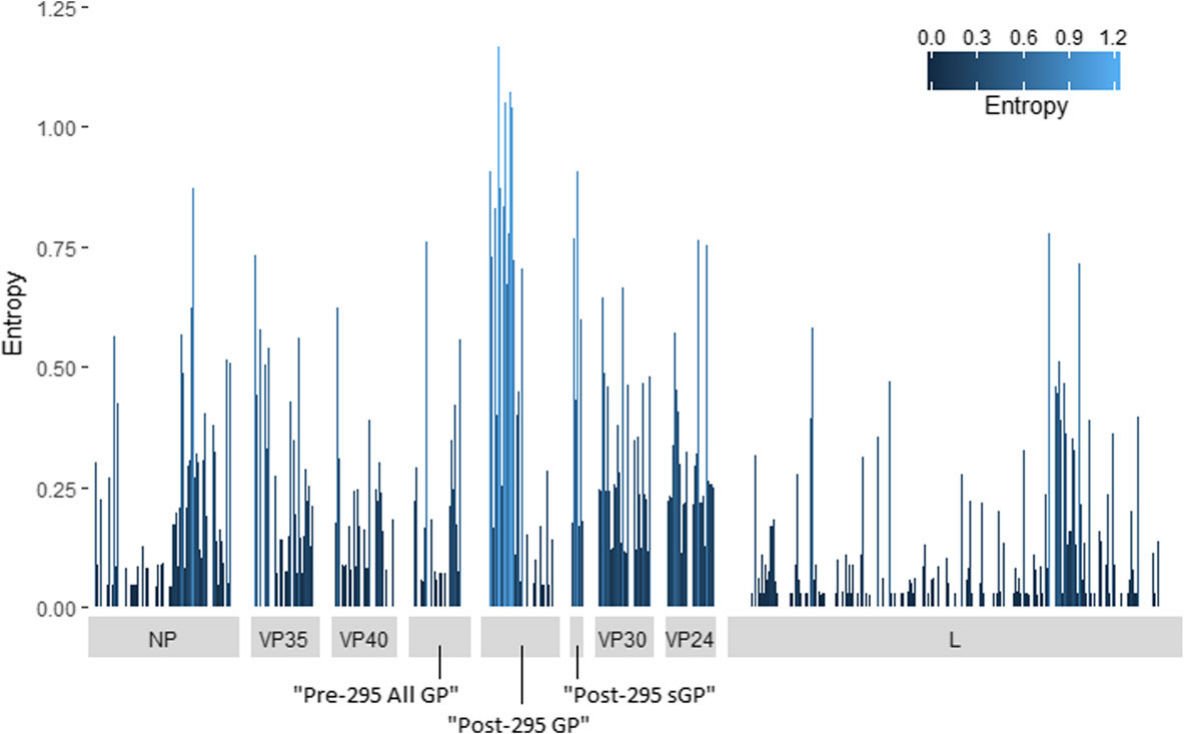
Protein sequence diversity of ZEBOV proteome. Shannon’s entropy was used as a general measure of protein sequence diversity for each aligned nonamer (nine amino acids) position (1–9, 2–10, etc.) of ZEBOV proteins. The x-axis represents the position along the length of the protein and the y-axis represents the entropy value, which is indicative of the level of variability at the corresponding nonamer positions, with a zero representing completely conserved sites and high entropy values of more than 1 marking diverse sites. “Post-295 ssGP” is not shown because it was not analysed (see Methods)

The entropy of the proteins ranged from 0 to ~1.2 (Fig. 1), with numerous regions of low entropy, indicating that the viral proteome is generally highly conserved (average proteome-wide entropy of 0.16; Additional file 1). Among the proteins, the most diverse was “Post-295 sGP” (average entropy of 0.51), while the most conserved was L (average entropy of 0.08). The “Post-295 GP” had the second highest average entropy (0.36), but included some of the most diverse positions of the proteome. Approximately 40% of the nonamer positions of L were completely conserved (zero entropy). The virion structural proteins (VP40, VP35, VP30, and VP24) generally had low entropy regions interspersed by relatively high entropy positions. The nucleoprotein (NP) and “Pre-295 All GP” showed a similar pattern, with relatively diverse positions at the N- and C-termini and low entropy regions in the centre.

### Experimentally validated HLA ligands and T-cell epitopes of ZEBOV

A total of 840 HLA ligands of ZEBOV, of which three are T-cell epitopes, have been experimentally mapped and reported in the IEDB (Additional file 2). All the nine ZEBOV proteins have been mapped of T-cell epitopes/HLA ligands (Table 2), and are chiefly reported for NP and “All GP”, and they thus appear to be most immunogenic; these two proteins are also most packed with reported epitopes over the length. As many as 43 HLA alleles have been investigated and of these 11 are representative alleles of the A2 (HLA-A*02:01, HLA-A*02:03, HLA-A*02:06, HLA-A*68:02, HLA-A*69:01), A3 (HLA-A*03:01, HLA-A*11:01, HLA-A*31:01), and B7 (HLA-B*07:02, HLA-B*35:01, HLA-B*51:01) supertypes studied herein. Notable alleles that have been validated by a large number of reported studies are the HLA-A*02:01 (A2 supertype), HLA-A*03:01 (A3), HLA-A*11:01 (A3) and HLA-B*07:02 (B7). None of the HLA ligands were tested for at least half of the representative alleles of the A2, A3, and B7 supertypes, however, 111 appeared to be promiscuous to two or more of the representative alleles of at least one of the three supertypes.

**Table 2:** Number of reported experimentally mapped human T-cell epitopes/HLA ligands of ZEBOV by protein and alleles

Protein/HLA allele^a^	NP	VP35	VP40	“All GP”	VP30	VP24	L	Total
HLA-A*01:01	29	–	–	15	1	2	3	50
HLA-A*02:01	412	–	2	362	1	2	9	788
HLA-A*02:03	–	–	–	–	–	–	1	1
HLA-A*02:06	–	–	–	–	–	–	1	1
HLA-A*03:01	116	–	2	94	1	2	1	216
HLA-A*03:19	–	–	–	–	–	–	1	1
HLA-A*11:01	145	–	1	132	1	–	1	280
HLA-A*23:01	–	–	–	–	–	–	2	2
HLA-A*24:02	289	–	1	272	–	1	4	567
HLA-A*24:03	1	–	1	–	–	1	5	8
HLA-A*26:01	2	–	1	3	–	1	5	12
HLA-A*26:02	–	–	–	–	–	–	2	2
HLA-A*26:03	–	–	–	–	–	–	1	1
HLA-A*30:01	–	–	–	–	–	–	2	2
HLA-A*31:01	–	1	–	3	1	–	2	7
HLA-A*32:07	–	–	–	1	–	–	–	1
HLA-A*32:15	–	–	–	1	–	–	–	1
HLA-A*68:02	–	–	–	–	–	–	3	3
HLA-A*68:23	–	–	–	1	–	–	–	1
HLA-A*69:01	–	–	–	–	–	–	2	2
HLA-B*07:02	75	1	2	76	1	2	4	161
HLA-B*08:01	70	–	1	44	1	1	–	117
HLA-B*15:01	144	3	4	85	1	3	7	247
HLA-B*15:17	–	–	–	–	–	–	3	3
HLA-B*15:42	–	–	1	1	–	–	2	4
HLA-B*27:05	1	–	1	2	1	1	2	8
HLA-B*35:01	4	–	3	1	2	1	9	20
HLA-B*39:01	2	–	–	1	–	1	4	8
HLA-B*40:01	4	–	2	5	1	2	4	18
HLA-B*45:06	–	–	1	1	–	–	2	4
HLA-B*46:01	–	–	–	–	–	–	1	1
HLA-B*51:01	1	–	–	–	–	–	–	1
HLA-B*57:01	–	–	–	–	–	–	1	1
HLA-B*58:01	2	1	1	3	1	1	5	14
HLA-B*83:01	–	–	1	1	–	–	2	4
HLA-C*03:03	1	–	–	1	–	–	1	3
HLA-C*04:01	–	–	1	1	–	–	2	4
HLA-C*05:01	–	–	–	–	–	1	–	1
HLA-C*06:02	1	–	–	–	–	–	2	3
HLA-C*07:02	–	–	–	–	–	–	1	1
HLA-C*12:03	–	1	–	–	–	–	–	1
HLA-C*14:02	–	–	–	–	–	1	1	2
HLA-C*15:02	–	–	–	–	–	–	1	1
TOTAL	1299	7	26	1106	13	23	99	2573

The number is larger than 840 HLA ligands reported because an allele was often studied for more than allele. ^a^ Representative alleles are in bold

### Potential T-cell epitopes and hotspots within ZEBOV proteome

**Fig. 2:**
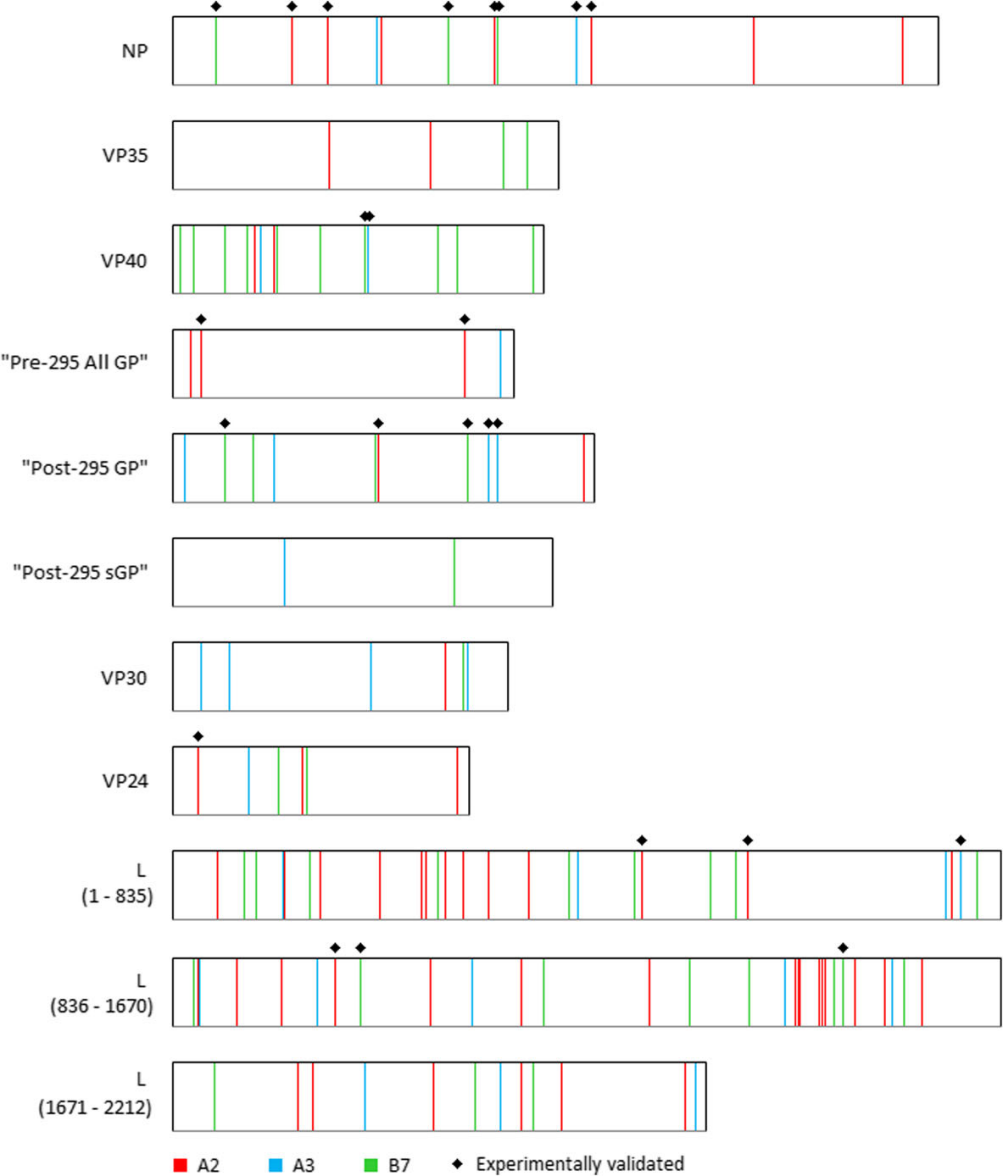
ZEBOV proteome map of putative HLA A2-, A3-, and B7-supertype-specific epitopes. The width of the boxes corresponds to the length of the proteins. Vertical lines represent the first amino acid of the putative epitopes and marked with * are such epitopes that overlapped experimentally validated HLA T-cell epitopes/HLA ligands

A total of 185 epitopes were predicted for the three HLA-supertypes: 82 (A2), 37 (A3) and 66 (B7) (Additional files 3 and 4). Notably, L was highly enriched (the percentage of the proteome-wide predicted epitopes that are found in a given protein; the number of epitopes found in a protein divided by the total number of proteome predicted epitopes, converted as a percentage) of the epitopes (~53%), while “Post-295 sGP” (~3%) had the least. Many of the epitopes were localized at the same position as the others, resulting in a reduction to 125 epitope positions (Fig. 2): NP (12), VP35 (4), VP40 (14), “Pre-295 All GP” (4), “Post-295 GP” (10), “Post-295 sGP” (2), VP30 (6), VP24 (6) and L (67). It was not L, but VP40 which was most packed (the percentage of the length of the protein spanned by the predicted epitopes over the total length of the protein) with epitopes over the protein length, while “Post-295 sGP” had the least.

Mapped epitope alignment positions generally showed low entropy, ranging from 0 at multiple locations to 0.93 (position 367 of GP). A majority (119/125) of the positions were highly conserved, with entropy <0.5 (Additional file 4). Eighty (80) of the alignment positions from “Post-295 GP”, VP30, and L corresponded to reported functional motifs/domains (Additional file 4). The structural localization of 38 of the epitopes from NP, VP35, VP40, “Pre-295 All GP”, “Post-295 GP”, VP 30 and VP24 was largely (24) partially exposed, 11 exposed and three buried at the surface of the corresponding PDB structures.

A number of the putative A2 supertype epitopes clustered to form hotspots: L protein, 1464-LLYSFGAFVSYYL-1476 and 1487-TLDNFLYYLTTQIHNL-1503. None of the putative epitopes were predicted for more than one supertype.

Twenty-four of the putative epitope sequences (12 completely conserved) matched reported experimentally validated HLA ligands/T-cell epitopes of A2, A3 and/or B7 supertype representative allele restrictions (Table 3). Eleven (11) of these putative epitopes were predicted for A2 supertype, five for A3 and eight for B7. At least one representative allele had been experimentally tested for each putative epitope, with as many as two (predicted for A2), five (A3), and four (B7) putative epitopes appearing to be promiscuous empirically for A2 (experimentally tested with two alleles), A3 (two alleles) and B7 (two or three alleles for a peptide) supertypes, respectively.

**Table 3:** Reported human T-cell epitopes/HLA ligands of ZEBOV that matched the predicted epitopes

Protein	Sequence	NetCTLpan supertype prediction	Antigen specific T-cell response
			HLA alleles assessed
NP	IPVYQVNNL	B7	B7: HLA-B*07:02
	RLEELLPAV	A2	A2: HLA-A*02:01
	FLSFASLFL	A2	A2: HLA-A*02:01
	HPLARTAKV	B7	B7: HLA-B*07:02, HLA-B*35:01, HLA-B*51:01
	GLFPQLSAI	A2	A2: HLA-A*02:01
	FPQLSAIAL	B7	A2: HLA-A*02:01 B7: HLA-B*07:02
	QTNAMVTLR	A3	A3: HLA-A*03:01, HLA-A*11:01
	KLTEAITAA	A2	A2: HLA-A*02:01
VP40	LPQYFTFDL	B7	B7: HLA-B*07:02, HLA-B*35:01
	FTFDLTALK	A3	A3: HLA-A*03:01, HLA-A*11:01
“Pre-295 All GP”	ILFQRTFSI	A2	A2: HLA-A*02:01 A3: HLA-A*03:01, HLA-A*11:01
	FLLQLNETI	A2	A2: HLA-A*02:01
“Post-295 GP”	MASENSSAM	B7	A2: HLA-A*02:01 B7: HLA-B*07:02
	LITNTIAGV	A2	A2: HLA-A*02:01
	LANETTQAL	B7	B7: HLA-B*07:02, HLA-B*35:01
	RTFSILNRK	A3	A2: HLA-A*02:01 A3: HLA-A*03:01, HLA-A*11:01
	KAIDFLLQR	A3	A2: HLA-A*02:01 A3: HLA-A*03:01, HLA-A*11:01
VP24	VLSDLCNFL	A2	A2: HLA-A*02:01
L	IISDLSIFI	A2	A2: HLA-A*02:01, HLA-A*69:01
	LLADGLAKA	A2	A2: HLA-A*02:01, HLA-A*69:01
	HSGFIYFGK	A3	A3: HLA-A*11:01, HLA-A*31:01
	KLINTLFHA	A2	A2: HLA-A*02:01
	TPVMSRFAA	B7	B7: HLA-B*07:02, HLA-B*35:01
	KPTFKHASV	B7	B7: HLA-B*07:02

### Antigenic diversity of putative HLA supertype-specific T-cell epitopes

**Fig. 3:**
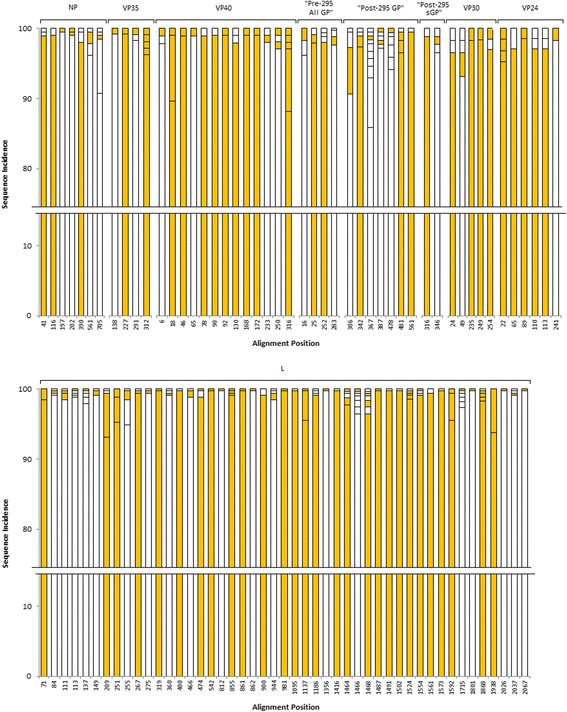
Antigenic diversity of mapped epitope alignment positions. Stacked bars were plotted based on the incidence rate (y-axis) of sequences at the alignment positions (x-axis). Colored in orange were the epitopes, while white were non-epitope sequences. Completely conserved epitope positions are not shown

Thirty (30) of the 125 mapped positions had a single putative epitope each that was completely conserved among all the sequences (100% incidence) and may be attractive for vaccine design (Table 4). The remaining (95) positions had one or more putative epitopes, with or without non-epitope variants (Fig. 3). The majority (57) of these exhibited a dominant putative epitope sequence (~88-99% incidence), while the other positions (38) comprised of only low incidence putative epitopes (< 1 - ~ 9% each), dominated by a single non-epitope variant (~86-99%), and often accompanied by other low incidence non-epitope variants (< 1 - ~8%).

**Table 4:** Candidate vaccine targets for ZEBOV. These are completely conserved sequences and predicted to be HLA supertype-restricted

Protein	Position	Epitope	Supertype specificity
NP	150	FLSFASLFL	A2
	265	HPLARTAKV	B7
	311	GLFPQLSAI	A2
	313	FPQLSAIAL	B7
	404	KLTEAITAA	A2
VP40	73	FILEAMVNV	A2
"Post-295 GP"	580	RTFSILNRK	A3
	588	KAIDFLLQR	A3
	667	ALFCICKFV	A2
VP30	171	LTLCAVMTR	A3
L	45	KLPKHIYRL	A2
	293	KIIKFLEPL	A2
	409	CVFKYSIAK	A3
	568	YPTRNVQTL	B7
	580	LLADGLAKA	A2
	780	TSACGIFLK	A3
	786	FLKPDETFV	A2
	795	HSGFIYFGK	A3
	999	KLINTLFHA	A2
	1024	TPVMSRFAA	B7
	1316	FQNVINYAV	A2
	1208	KPKCPSAAL	B7
	1453	TTHFLTYPK	A3
	1494	YLTTQIHNL	A2
	1511	KPTFKHASV	B7
	1816	KLDEVLWEI	A2
	1979	APFFATGYL	B7
	2004	LTNFLSTTR	A3
	2192	KLIERLTGL	A2
	2202	SLFPDGLYR	A3

**Fig. 4:**
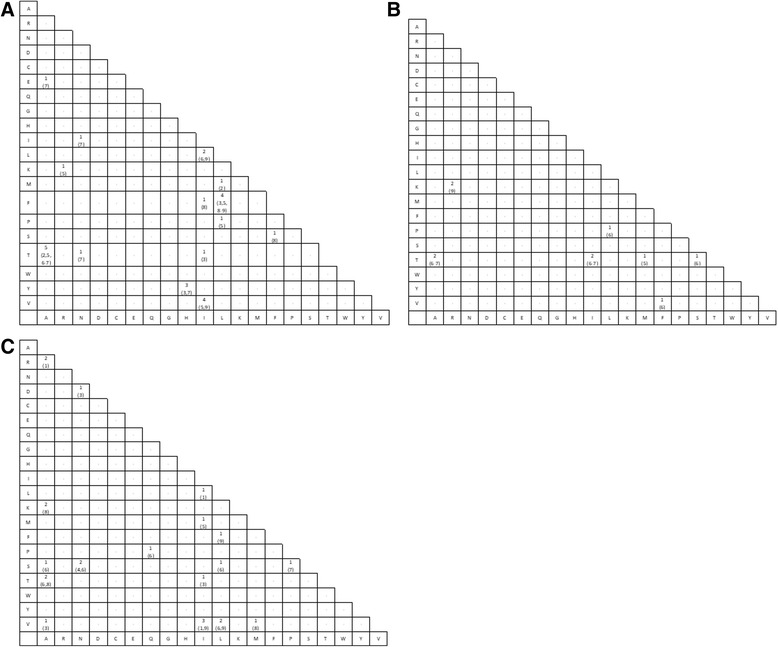
Matrix of amino acid substitutions observed within the putative epitope peptides of A2 (panel **a**), A3 (panel **b**) and B7 (panel **c**) supertypes. Only positions that had more than one putative epitope were considered; the dominant (% incidence) among the putative epitope sequences at the position served as the reference to identify the mutations. Numbers: Substitution frequency, with the peptide amino acid position where the substitution occurred shown in bracket; Dash “-”: No substitution

Thirty-nine (39) of the 125 epitope positions had more than one putative epitope to allow comparison of acceptable substitution (i.e. positions with a completely conserved epitope or only one epitope were not included in this analysis). Such positions had two to as many as five epitope sequences (only two positions had five: VP35_312_ and VP40_316_); the dominant (% incidence) among the putative epitope sequences at the position served as the reference sequence. A total of 46 distinct amino acid substitutions (irrespective of the amino acid positions in the nonamer) were observed between the putative epitopes at these positions (Additional file 4; Fig. 4): 18 for A2, 8 for A3 and 20 of B7. All of the epitopes exhibited only one amino acid substitution in the sequence, except seven that had two substitutions each over the epitope length and six with at least three substitutions each over the epitope length (Additional file 5). The most common substitution was between Alanine (A) and Threonine (T) for A2, Alanine (A) and Threonine (T), Arginine (R) and Lysine (K), and Isoleucine (I) and Threonine (T) for A3, and Isoleucine (I) and Valine (V) for B7 (Fig. 4). Peptide amino acid positions with the most number of substitution were position 6 (14 substitutions), followed by position 7 and 9 (both with 10 substitutions) (Additional file 5). Position 2 showed only two substitutions (Leucine (L) to Methionine (M) and Alanine (A) to Threonine (T)) for A2 supertype (Fig. 4), and none for the others (although no substitution, the following amino acids were observed at position 2 (Additional file 5): Threonine (T)/Alanine (A)/Leucine (L)/Glutamine (Q)/Valine (V)/Serine (S) for A3 and Proline (P)/Alanine (A) for B7). Several of the putative epitopes were tolerant to multiple amino acid substitutions. Notably, position 312 of VP35 and 316 of VP40 each had a dominant B7 putative epitope sequence with four other sequences, where each had at least one amino acid difference to the dominant putative epitope, but all were predicted to be B7 supertype epitopes despite the antigenic variation.

## Discussions

In this study, we mapped and analysed putative HLA-A2, -A3, and -B7 supertype-restricted T-cell epitopes of ZEBOV, the most pathogenic species among the EBOV family. The large number of ZEBOV sequences (14,098) analysed, isolated from different geographical regions of Africa and from as early as 1992, offered information for a broad survey of EBOV protein diversity in nature and their relevance as targets of immune responses. Although a large number of ZEBOV sequences are reported in the NCBI Protein Database, only ~9% were non-redundant. The redundant sequences were present in each of the protein datasets at about relatively similar levels, with no specific preference for any of the protein. Analysis of the duplicate sequence records showed difference in data for the geographical location, isolate and/or biosample fields. This is likely a result of ebola surveillance programs that end up identifying largely identical or highly similar circulating isolates. Although to some extent the redundancy may be accepted as a reflection of the incidence of the corresponding EBOV isolates in nature, the analysis was performed on the non-redundant dataset to minimize sampling bias. However, this introduces potential bias in the entropy values. Removal of duplicate protein sequences will reduce the number of nonamer sequences for all positions of the proteome, and thus increasing the entropy value of each position, the degree of which depends on the number of duplicates removed. The entropy bias was minimized through the correction to the estimation of entropy for large data size.

Entropy analysis revealed that ZEBOV proteome is generally conserved, despite the relatively long evolutionary history of the virus, with reported data of isolates between 1992 and 2016. The lethal nature of the virus to the host and the limited geographical spread of the virus may be contributing factors to the low variability of the virus. As a comparison, the diversity of ZEBOV proteome (max. Entropy of 1.2) is on the lower side compared to other RNA viruses, such as West Nile virus (max. Entropy of ~2) [42], dengue virus (4 subtypes; max. Entropy of ~4) [41], influenza A virus (subtype: H1N1; max. Entropy ~3) [43] and human immunodeficiency virus 1 (clade B; max. Entropy of ~9) [52]. Like most RNA viruses, the functionally critical L protein, which serves as the replication agent (polymerase) of the virus [53], was most conserved, while the surface glycoprotein GP, which mediates viral entry into the host [53] and is target of immune recognition [53], was most diverse.

The availability of reliable computational tools with good reported prediction capability enable systematic screening of candidate T-cell epitopes from larger sets of protein antigens, such as those encoded by complete viral genomes. Putative T-cell epitopes for HLA-A2, -A3, and -B7 supertypes were mapped onto the aligned non-redundant sequences of each ZEBOV protein. Notably, it was observed that the putative epitopes had a propensity to co-localize at select positions of the protein multiple sequence alignment. This preferential localization of the epitopes indicates that they are structural in nature, though the individual epitope sequences may vary (i.e. structure conservation maintained). A similar observation has been made in other studies, such as localization of T-cell epitope clusters to exposed strands of HIV envelope glycoprotein (clades B and D) [54] and localization of myoglobin T-cell epitopes at the N-terminus of different myglobin fragments [55]. This was also observed extensively among the sequences of dengue serotypes, the preferential localization of which even extended to other *flaviviruses* [56]. A likely explanation of such an extensive conserved localization may be that the physical location of the peptide within the native protein leads to differential antigenic processing and consequent epitope selection [54]. Preferential localization of epitopes across multiple aligned sequences of a protein of a viral species, which extends to variant sequences of homologous proteins of other species with similar genomic architecture has important implications for epitope-based vaccine design and merits further investigation. This is because the conserved localization may allow design of vaccines that target multiple viral species and provide broad coverage of variants within each species, but may also be a cause for concern of altered-peptide ligand effects [57]. Additionally, the preferential localization can facilitate mapping of immune targets in novel variants by focusing on such regions.

Experimental measurements as validation of computational predictions are necessary for accurate interpretation of results. Computational models that are valid, relevant, and properly assessed for accuracy are useful for planning of complementary laboratory experiments [17, 58]. The prediction system NetCTLpan, which was used herein to predict HLA-A2, -A3, and -B7 supertype-restricted epitopes has been trained and rigorously tested using experimentally known peptides [45]. The tool takes an integrative approach of modelling various aspects of the antigen processing and presentation pathway, and the predictive performance has been shown to surpass other state-of-the-art class I epitope prediction methods. Experimentally validated T-cell epitopes/HLA ligands reported in the public database allow assessment of the reliability of the predictions. Although a large number (840) of human T-cell epitopes/HLA ligands of ZEBOV have been reported, none of them were experimentally tested for more than half of the supertype representative alleles. Nonetheless, 11 of the putative epitopes appeared to be promiscuous empirically for at least one of the three supertypes.

All the nine ZEBOV proteins have been experimentally mapped of T-cell epitopes/HLA ligands, with a majority reported for NP and “All GP” (primarily the structural GP; not soluble sGP and small soluble ssGP). The putative HLA-A2, -A3, and -B7 supertype-restricted epitopes were predicted herein for each of the ZEBOV proteins. In contrast to the experimental data, which did not include any supertype-restricted epitopes, L was highly enriched and VP40 was most packed with putative epitopes over the protein length. The structural GP has become the main focus in many research efforts as it is the only known protein on the surface of the virus, which is presumed to mediate the viral entry into host cells [53]. Thus, interfering with the viral entry into the host cells may be the earliest protection step against EBOV. Non-structural, soluble glycoprotein, sGP, is proposed to be an efficient binder onto antibodies capable of recognizing membrane-bound GP. The major ribonucleoprotein, NP, aggregates with VP30 to form a complex with VP35 and L which then interacts with the viral genomic RNA to create the nucleocapsid of the EBOV. The proteins VP35 and L are responsible for the transcription and replication of the EBOV genome [53]. The VP35 is an interferon (IFN) antagonist that blocks the induction of the antiviral immune response [53]. The VP40 plays an important role in the maturation of the virus through the virion assembly process and is believed to be a potent inhibitor of type I interferon response [53, 59]. Thirty (30) of the mapped epitope positions in NP, Post-295 GP, L and VP40 had a single epitope that was completely conserved among all the viral sequences (100% incidence). In fact, a majority of the positions were highly conserved, with an entropy <0.5; a number of them corresponded to sites critical to viral structure-function and thus are likely to be evolutionarily robust. These sequences are candidates for consideration of rational epitope-based vaccine design [60, 61], applicable for the general population and effective against a spectrum of ebola variants. The adaptive immune response (both humoral and cellular) plays a key role in protection against viral pathogens [6]. The cellular T-cell responses, involving both the CD4^+^ and CD8^+^ T cells, have been implicated to be important in providing protection against the EBOV [7]. Immunization with adenoviral vectors (AdV) encoding the GP and NP stimulated the induction of effective protection in nonhuman primates [8].

Thirty-nine (39) of the 125 epitope positions had more than one epitope to allow a comparison of acceptable substitution. A total of 46 distinct amino acid substitutions were observed between the putative epitopes at these positions: 18 for A2, 8 for A3 and 20 of B7. Several of the putative epitopes were tolerant to multiple amino acid substitutions. The amino acid substitutions reported herein at the critical peptide positions 2 and those in the C-terminus are in agreement with the amino acids recognised by the representative alleles of each of the supertypes [62], however the band of antigenic change that did not abrogate peptide recognition was narrower for ZEBOV. Analysis of ZEBOV proteins with an available 3D structure showed no correlation between conservation and localization of the epitopes (buried, partial and exposed), however, the three epitopes observed to be buried were completely conserved. Given the overall low variability of the virus and that the substitutions observed appeared to be conservative in terms of recognition by the supertype alleles, suggests that epitopes are likely to remain antigenically conserved in general, with a low likelihood of immune escape.

The EBOV is markedly one of the most fatal pathogen and thus far, there are no effective vaccines or therapeutic measures against them. The data herein offers insights into ZEBOV diversity, its evolutionary history, and provides a catalogue of mapped epitopes (experimental and putative), matrix of acceptable epitope substitutions, and candidates for rational vaccine design, which are also attractive for a structure-based design of candidate inhibitory compounds, and improvement of the current diagnostic methods.

## Additional files

Additional file 1: Figure S1. Average proteome entropy of each ZEBOV protein and the complete proteome. The entropy values for each protein were 0.16 (NP), 0.21 (VP35), 0.15 (VP40), 0.15 (“Pre-295 All GP”), 0.36 (“Post-295 GP”), 0.51 (“Post-295 sGP”), 0.29 (VP30), 0.30 (VP24) and 0.08 (L). “Post-295 ssGP” is not shown because it was not analysed (see Methods). The average proteome entropy was 0.16. (TIFF 81 kb)

Additional file 2: Table S1. Reported human T-cell epitopes and HLA ligands of ZEBOV from the Immune Epitope Database and Analysis Resource (IEDB; as of January 2017). (TIFF 33 kb)

Additional file 3: Table S2. Number of putative HLA-A2, -A3, and -B7 supertype-restricted epitopes of ZEBOV. (TIFF 163 kb)

Additional file 4: Table S3. Sequence diversity at each epitope position. (TIFF 142 kb)

Additional file 5: Table S4. Substitutions observed between putative epitopes. Only shown for positions that had more than one putative epitope, to allow comparison of acceptable substitution. (PDF 229 kb)

## Abbreviations

AdV: Adenoviral vectors
EBOV: *Ebolavirus*
EVD: Ebola virus disease
GP: Glycoprotein
HLA: Human leukocyte antigen
IEDB: Immune Epitope Database and Analysis Resource
L: RNA-dependent RNA polymerase
NP: Nucleoprotein
sGP: Non-structural soluble glycoprotein
ssGP: Second secreted glycoprotein
VP24: Membrane associated protein
VP30: Transcription factor
VP35: Polymerase cofactor
VP40: Matrix protein
ZEBOV: *Zaire ebolavirus*
