# Supplementary figures and images for: Mapping HLA-A2, -A3 and -B7 supertype-restricted T-cell epitopes in the ebolavirus proteome
Source: BMC Genomics. 2018 Jan 19;19(Suppl 1):42. doi: 10.1186/s12864-017-4328-8 (PMC5780746; doi:10.1186/s12864-017-4328-8)

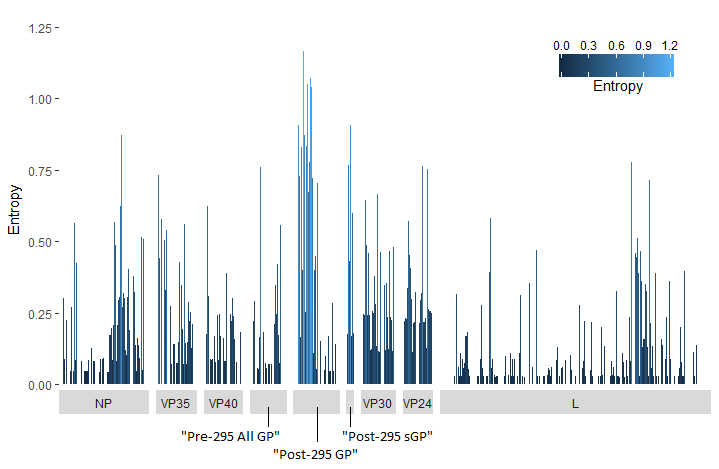

Supplement: Supplementary file 1 — Average proteome entropy of each ZEBOV protein and the complete proteome. The entropy values for each protein were 0.16 (NP), 0.21 (VP35), 0.15 (VP40), 0.15 (“Pre-295 All GP”), 0.36 (“Post-295 GP”), 0.51 (“Post-295 sGP”), 0.29 (VP30), 0.30 (VP24) and 0.08 (L). “Post-295 ssGP” is not shown because it was not analysed (see Methods). The average proteome entropy was 0.16. (TIFF 81 kb) [file 12864_2017_4328_MOESM1_ESM.tif]

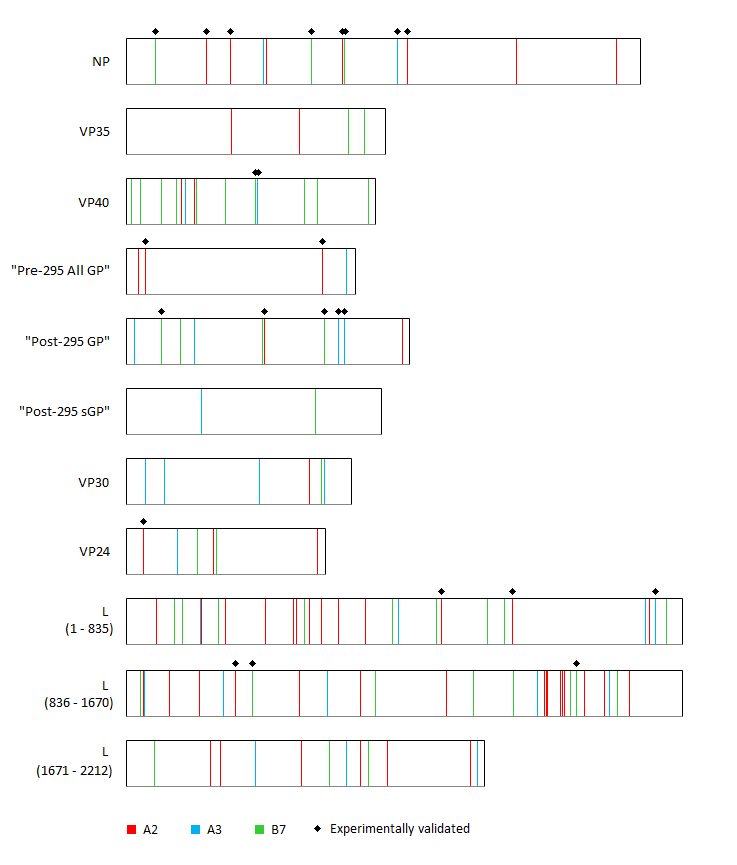

Supplement: Supplementary file 2 — Reported human T-cell epitopes and HLA ligands of ZEBOV from the Immune Epitope Database and Analysis Resource (IEDB; as of January 2017). (TIFF 33 kb) [file 12864_2017_4328_MOESM2_ESM.tif]

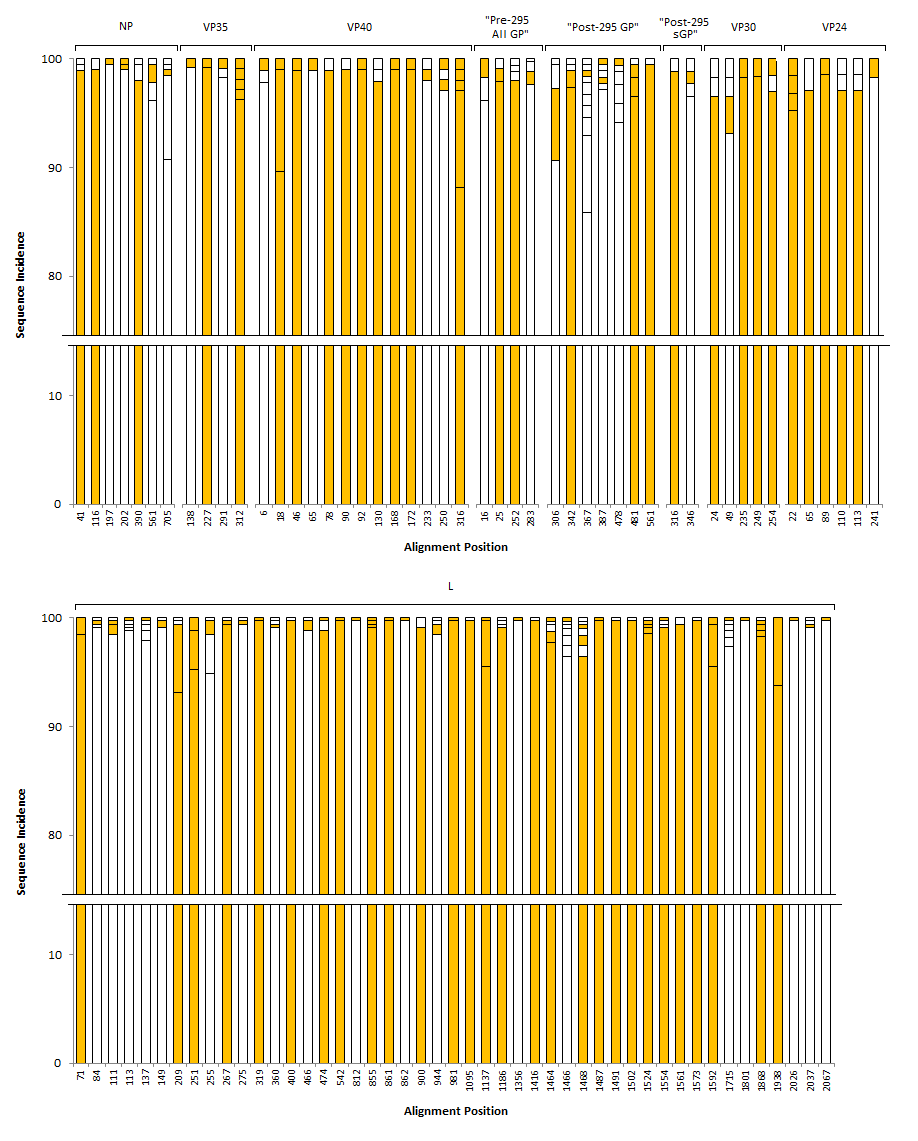

Supplement: Supplementary file 3 — Number of putative HLA-A2, -A3, and -B7 supertype-restricted epitopes of ZEBOV. (TIFF 163 kb) [file 12864_2017_4328_MOESM3_ESM.tif]

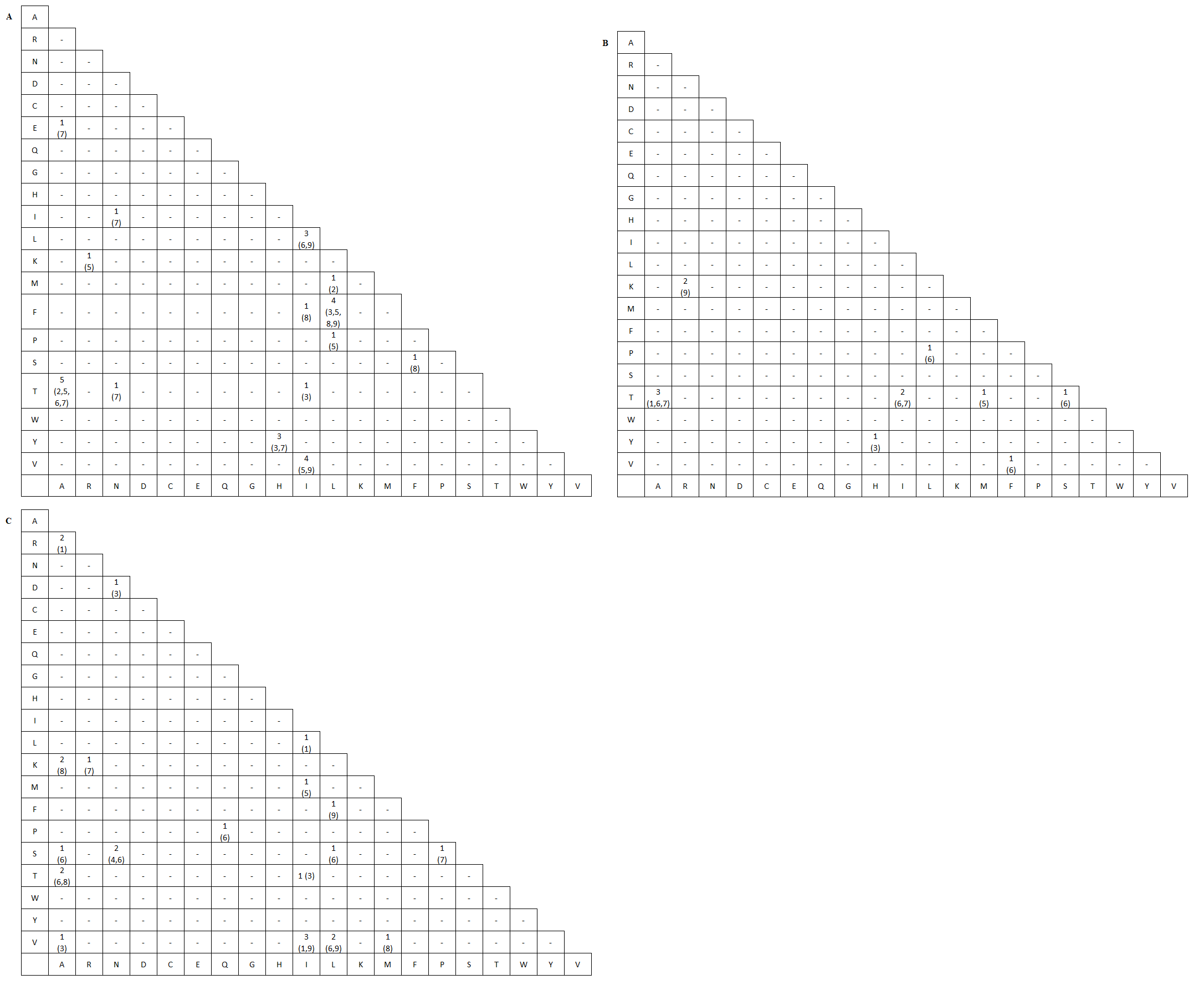

Supplement: Supplementary file 4 — Sequence diversity at each epitope position. (TIFF 142 kb) [file 12864_2017_4328_MOESM4_ESM.tif]
