# Supplementary material for: Mapping HLA-A2, -A3 and -B7 supertype-restricted T-cell epitopes in the ebolavirus proteome
Source: BMC Genomics. 2018 Jan 19;19(Suppl 1):42. doi: 10.1186/s12864-017-4328-8 (PMC5780746; doi:10.1186/s12864-017-4328-8)
Supplement: Supplementary file 5 — Substitutions observed between putative epitopes. Only shown for positions that had more than one putative epitope, to allow comparison of acceptable substitution. (PDF 229 kb) [file 12864_2017_4328_MOESM5_ESM.pdf]

Additional File 5: Table S4 Substitutions observed between putative epitopes. Only shown for positions that had more than one putative epitope, to allow comparison of acceptable substitution.

1 substitution (26 positions)			
Protein	Position	Peptide	Supertype restriction
NP	202	RLMRTNFLL	A2
		.M.......	
VP35	227	IMYDHLPGF	A2
		L	
VP40	46	TPSNPLRPI	B7
		...S.....	
	92	IPIWLPLGV	B7
		..T......	
	168	LPQYFTFDL	B7
		I........	
	172	FTFDLTALK	A3
		.....A...	
	250	VPIDPTRNI	B7
		I........	
Pre-295 All GP	25	ILFQRTFSI	A2
		....K....	
Post-295 GP	342	MASENSSAM	B7
		.......T.	
	387	STYNTPVYK	A3
		.....L...	
VP30	235	LIMFITAFL	A2
		.....A...	
	249	LPCESSAVV	B7
		.....A...	
	254	SAVVVSGLR	A3
		.....T...	
VP24	89	SPLWALRVI	B7
		.....V...	
L	71	LPIDFIVPI	B7
		V	
	111	TLFLKYYLK	A3
		........R	
	209	AMDWYQTSV	A2
		......A..	
	267	YPNFKIVSM	B7
		..D......	
	319	FLTQMHLAV	A2
		..I......	
	474	IISDLSIFI	A2
		V	
	542	LPQYRNFSF	B7
		.....S...	
	861	AAFHTFFSV	A2
		..L......	
	981	TSFLRQIVR	A3
		......T..	
	1487	TLDNFLYYL	A2
		.....I...	
	1554	SISSFLTFV	A2
		......A..	
	1938	KLYEAVHKL	A2
		......Y..	

2 substitutions (7 positions)			
Protein	Position	Peptide	Supertype restriction
VP40	18	YPARSNSTI	B7
		..V......	
		......P..	
Post-295 GP	481	LITNTIAGV	A2
		..A......	
		....A....	
L	251	TLISKIAEV	A2
		I	
		.....L...	
	1137	EQFKVVWLK	A3
		.....F...	
		........R	
	1464	LLYSFGAFV	A2
		.......L.	
		.......I.	
	1468	FGAFVSYYL	A2
		......H..	
		....I....	
	1592	FLHQIVELL	A2
		..Y......	
		......A..	

More than 2 substitutions (6 positions)			
Protein	Position	Peptide	Supertype restriction
VP35	312	RPVPPSPKI	B7
		.....L...	
		.......A.	
		A........	
		A......A.	
VP40	316	SPASLPAVV	B7
		I	
		L	
		.....Q...	
		.......M.	
VP24	22	VLSDLCNFL	A2
		......I..	
		....F....	
		......T..	
L	855	FPCRITAAF	B7
		.....A...	
		L	
		....M....	
	1524	MSIDPHFSI	A2
		.......F.	
		....L....	
		V	
	1868	IVSGMTTPR	A3
		....T....	
		......A..	
		.....I...	
